# Prognostic utility of combined 256-detector-row computed tomographic pulmonary angiography and D-dimer in risk stratification of acute pulmonary embolism: a retrospective cohort analysis

**DOI:** 10.3389/fphys.2026.1796105

**Published:** 2026-04-10

**Authors:** Yanxian Li, Jun Sang, Xuemei Li, Wenjing Ding, Chunyan Yang, Zheng Yang, Shiping Zhao, Yanfang Liu

**Affiliations:** 1 Baoshan College of Traditional Chinese Medicine, Baoshan, Yunnan, China; 2 Department of Medical Imaging, Baoshan People’s Hospital, Baoshan, Yunnan, China

**Keywords:** acute pulmonary embolism, computed tomographic pulmonary angiography, D-dimer, prognosis, right ventricular/left ventricular diameter ratio, risk stratification

## Abstract

**Objective:**

The aim of this study was to assess the predictive value of 256-detector-row helical computed tomographic pulmonary angiography (CTPA) parameters, combined with serum D-dimer levels, for risk stratification and 30-day prognostic assessment in patients diagnosed with acute pulmonary embolism (APE).

**Methods:**

This retrospective cohort study included clinical data from 77 patients with APE, stratified into low-risk (n = 38), intermediate-low-risk (n = 17), intermediate-high-risk (n = 14), and high-risk (n = 8) groups, as well as 89 control individuals, treated at Baoshan People’s Hospital between January 2021 and December 2024. Clinical characteristics, CTPA-derived metrics [pulmonary artery (PA)-to-ascending aorta diameter ratio, right-to-left ventricular diameter ratio (RVD/LVD), and superior vena cava diameter], and D-dimer levels were analyzed and compared across groups. Receiver operating characteristic curve analysis and multivariate logistic regression were used to assess predictive performance.

**Results:**

The incidence of bilateral pulmonary embolism increased with escalating risk stratification, from 44.7% in the low-risk group to 87.5% in the high-risk group. Similarly, the frequency of thrombus involvement in the main PA and its branches rose from 28.9% to 100%. The RVD/LVD ratio demonstrated a significant positive correlation with risk stratification (ρ = 0.492;95%CI: 0.30–0.64; *p* < 0.001) and showed statistically significant differences across groups (H = 26.093, *p* < 0.001); median values were higher in the intermediate-high-risk (1.55) and high-risk (1.52) groups compared with the low-risk group (0.99). D-dimer concentrations increased progressively with risk stratification (H = 45.593, *p* < 0.001) and effectively differentiated patients with APE from control individuals (*p* < 0.001), although no significant differences were observed among APE subgroups. The combined model incorporating RVD/LVD and D-dimer yielded an area under the curve (AUC) of 0.917 (95% CI: 0.852–0.983) for predicting high-risk APE, surpassing the performance of individual indicators (RVD/LVD AUC = 0.874; D-dimer AUC = 0.716). Multivariate logistic regression identified both RVD/LVD (OR = 124.605, *p* = 0.001) and D-dimer (OR = 1.313, *p* = 0.032) as independent predictors of poor short-term prognosis.

**Conclusion:**

The integration of CTPA-derived imaging parameters with D-dimer levels shows promise for risk stratification and prognostic assessment in APE, with the RVD/LVD ratio emerging as a key imaging biomarker. However, given the single-center retrospective design and limited sample size, these findings should be considered exploratory. The combined model requires external validation in larger, multicenter cohorts before its routine clinical applicability, especially in diverse healthcare settings, can be established.

## 1 Introduction

Acute pulmonary embolism (APE) is a major cause of in-hospital mortality and represents the third most common cause of cardiovascular-related death (Roy et al., 2022). The clinical presentation among patients is highly heterogeneous, ranging from asymptomatic manifestations to hemodynamic instability and death. Therapeutic strategies, including anticoagulation, thrombolysis, and interventional procedures, are guided by risk stratification; therefore, timely and accurate diagnosis, together with precise risk assessment, are essential for improving clinical outcomes (Friedman et al., 2024).

Currently, computed tomographic pulmonary angiography (CTPA) is considered the gold standard for the diagnosis of APE, providing reliable anatomical and functional information (Lanzafame et al., 2025). When compared with conventional 16-detector-row or 64-detector-row computed tomography (CT), 256-detector-row helical CT provides sub-second temporal resolution and submillimeter spatial resolution, enabling detailed visualization of thrombi within the pulmonary artery (PA) subsegmental branches and more distal vessels. The use of low-dose scanning protocols (tube voltage 80–100 kV with automatic tube current modulation) reduces radiation exposure, while advanced post-processing techniques, including volume rendering and multiplanar reconstruction, improve the accuracy of right ventricular functional assessment (Martinez Licha et al., 2020; Wanf and Xu, 2020). However, dependence on a single CTPA-derived parameter does not fully capture the underlying pathophysiological changes associated with APE, thereby limiting a comprehensive assessment.

D-dimer, a fibrin degradation product, functions as a sensitive marker of systemic fibrinolytic activity. Its diagnostic sensitivity for APE exceeds 90%, supporting its use in exclusionary diagnostic assessment. However, its specificity remains limited (approximately 40%), as elevated D-dimer levels may occur in the context of malignancy, infection, or postoperative states, thereby limiting its standalone value for risk stratification and prognostic evaluation in APE (Feng and Wu, 2021). The 2019 European Society of Cardiology (ESC) guidelines recommend that risk assessment in APE incorporate clinical presentation, imaging indicators of right ventricular dysfunction, and serum biomarkers, emphasizing that reliance on a single parameter is insufficient for accurate stratification (Konstantinides et al., 2020).

Most prior investigations have focused on the combined application of 64-detector-row CTPA and D-dimer, although these studies have demonstrated limited accuracy in assessing right heart morphology. Additionally, the effective integration of CTPA-derived imaging parameters with D-dimer into a comprehensive predictive model has been infrequently achieved. In this context, this exploratory study systematically assessed the combined predictive value of multiple parameters obtained from 256-detector-row helical CTPA and D-dimer. Our primary aim was to develop and internally validate a preliminary model for risk stratification and prediction of 30-day poor prognosis in APE. We explicitly acknowledge that due to the retrospective nature and sample size constraints of this single-center study, the goal is to generate hypotheses and assess feasibility for future large-scale validation, rather than to establish immediate clinical guidelines. This approach addresses a significant gap in the literature and provides a potential foundation for developing a more robust, multimodal framework for risk assessment pending further confirmation.

## 2 Materials and methods

### 2.1 Study participants

This retrospective cohort study was approved by the Ethics Committee of Baoshan College of Traditional Chinese Medicine (Approval No.: BSZYZLL2023014). The requirement for informed consent was waived due to the retrospective design and use of de-identified medical records. A total of 77 patients diagnosed with APE via 256-detector-row helical CTPA at Baoshan People’s Hospital were consecutively enrolled as the case group between January 2021 and December 2024. Diagnosis and risk stratification were conducted in accordance with the 2019 ESC Guidelines and 2018 China Guidelines for the Diagnosis, Treatment, and Prevention of Pulmonary Thromboembolism (Wang, 2018; Konstantinides et al., 2020). Risk stratification was based on the presence of hemodynamic instability (shock or hypotension), right ventricular dysfunction (RVD), and elevated cardiac biomarkers. RVD was defined by specific imaging findings on echocardiography or CTPA (e.g., right ventricular dilation with RV/LV ratio > 1.0 or 0.9, right ventricular hypokinesis). Cardiac biomarkers included troponin and BNP/NT-proBNP. Based on these integrated criteria, patients were categorized into low-risk (n = 38), intermediate-low-risk (n = 17), intermediate-high-risk (n = 14), and high-risk (n = 8) groups. During the same period, 89 patients who presented with symptoms such as dyspnea and chest pain but were subsequently excluded from APE based on CTPA findings were selected as the control group. No statistically significant differences in baseline characteristics, including age and sex, were identified between the two groups (*p* > 0.05), indicating baseline comparability.

Inclusion criteria: 1) A diagnosis of APE consistent with the criteria outlined in the aforementioned guidelines; 2) age between 18 and 80 years; 3) A first episode of APE, with CTPA and D-dimer testing performed within 24 hours of hospital admission and no prior administration of anticoagulant or thrombolytic therapy; 4) Availability of complete clinical data.

Exclusion criteria: 1) Chronic pulmonary embolism or other pulmonary disorders, including chronic obstructive pulmonary disease or lung cancer; 2) Presence of severe cardiac insufficiency (New York Heart Association [NYHA] class IV), hepatic insufficiency (Child–Pugh class C), renal insufficiency (serum creatinine > 442 μmol/L), or advanced malignancy at the time of admission; 3) Incomplete medical records.

Definition of poor prognosis: Poor prognosis was defined as the occurrence of serious clinical events attributable to APE within 30 days of admission, including all-cause mortality, cardiopulmonary resuscitation, tracheal intubation with mechanical ventilation, systemic hypotension or shock requiring vasopressor support (dose > 5 μg/kg·min), or the administration of reperfusion therapies such as PA thrombectomy or thrombolysis.

### 2.2 Clinical data and study methods

#### 2.2.1 Clinical data collection

Clinical data were independently extracted by two investigators who were blinded to group allocation, using the electronic medical record system of the hospital. The collected variables included baseline characteristics (age, sex); personal history [smoking history (≥ 1 cigarette/day for ≥ 1 year), alcohol consumption history (≥ 20 g/day for ≥ 1 year)]; past medical history (hypertension, prior stroke, diabetes, history of lower extremity deep venous thrombosis [DVT]); and presenting clinical manifestations (dyspnea, chest pain, or hemoptysis).

#### 2.2.2 Plasma D-dimer detection

Upon admission, 2 mL of venous blood was collected from the antecubital vein of each individual, anticoagulated with sodium citrate, and analyzed for D-dimer using an immunoturbidimetric assay (analyzer: STA-GO MAX; reagent kit: Shanghai Sun Biotech Co., Ltd.). The reference range was 0–0.5 μg/mL.

#### 2.2.3 256-Detector-row helical CTPA examination and imaging analysis

A GE Revolution 256-detector-row helical CT scanner was used for all examinations. Scanning parameters were set as follows: tube voltage, 100 kV; tube current, 100–300 mA; slice thickness, 0.625 mm; and reconstruction interval, 0.625 mm. An iodinated contrast agent (iohexol, 350 mgI/mL, 50 mL), followed by 30 mL of saline, was administered intravenously via the antecubital vein at an injection rate of 3.5–5.0 mL/s, resulting in a total injection volume of 80 mL. Intelligent tracking was used to determine the scan delay, with image acquisition initiated once the CT attenuation value of the main PA reached 70 Hounsfield units. Source images were transferred to a General Electric Advantage Workstation post-processing workstation and independently processed and measured by two senior radiologists, each with more than 10 years of experience in thoracic imaging, using a double-blind method. In instances of disagreement, consensus was achieved through adjudication by a third senior radiologist. Measurement reliability was assessed using the intraclass correlation coefficient (ICC), with key parameters demonstrating ICC values exceeding 0.85. Imaging parameters and measurement methods were defined as follows:

1. Thrombus distribution: The involvement of pulmonary lobes (left lung, right lung, or bilateral lungs) and the level of affected vessels (main PA only, branch vessels only, or combined involvement of the main PA and its branches) were qualitatively documented.
2. Main PA/ascending aorta diameter ratio (PA/AO): On axial images obtained at the level of the PA bifurcation, the maximum internal diameters of the main PA and the AO at the same anatomical level were measured, and the ratio was subsequently calculated.
3. Right ventricular diameter/left ventricular diameter ratio (RVD/LVD): On the four-chamber cardiac view, the maximum short-axis internal diameters of the right ventricle (RVD) and left ventricle (LVD), measured perpendicular to the ventricular long axis, were obtained and used to calculate the ratio.

Superior vena cava diameter (SVC): The internal diameter of the superior vena cava was measured at its widest segment (Wang et al., 2022; Guo et al., 2025).

**Figure 1:**
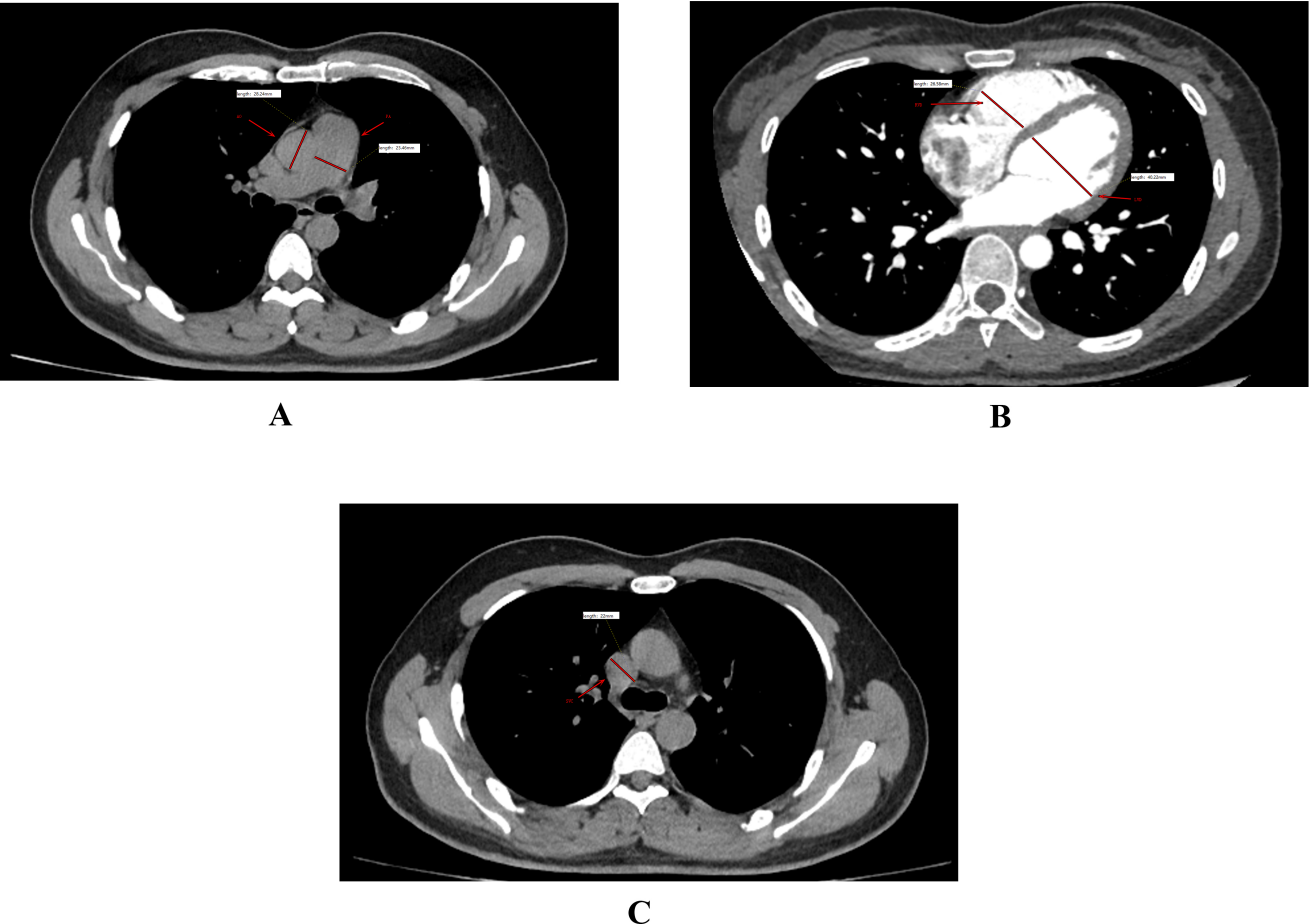
Schematic depiction of imaging parameter measurements in patients with APE. **(a)** Axial image depicting measurement of the internal diameters of the main PA and AO; **(b)** Four-chamber cardiac view illustrating measurement of the internal diameters of the RVD and LVD; **(c)** Measurement of the internal diameter at the widest segment of the SVC.

A schematic depiction of the imaging parameter measurements is presented in **Figure 1**.

### 2.3 Statistical analysis

Statistical analyses were conducted using SPSS version 27.0. The Shapiro–Wilk test was used to assess the normality of quantitative variables. Variables following a normal distribution were presented as mean ± standard deviation (mean ± SD), and intergroup comparisons were conducted using independent-samples t tests for two groups or one-way analysis of variance for comparisons among multiple groups. For non-normally distributed variables, data were expressed as median (lower quartile, upper quartile) [M (Q1, Q3)], with comparisons carried out using the Mann–Whitney U test for two groups or the Kruskal–Wallis H test for multiple groups. Categorical variables were summarized as counts and percentages [*n* (%)], and comparisons were performed using the chi-square test or Fisher’s exact test when more than 20% of expected cell counts were < 5. Correlations between D-dimer levels, CTPA parameters, and risk stratification were assessed using Spearman’s rank correlation coefficient (ρ). To enhance the robustness of the correlation estimates and address potential non-normality, 95% confidence intervals (CIs) for Spearman’s ρ were calculated using a non-parametric bootstrap procedure with 1,000 resamples. Predictive performance was assessed through the construction of receiver operating characteristic (ROC) curves, with calculation of the area under the curve (AUC), optimal cutoff values, sensitivity, specificity, positive predictive value, and negative predictive value (NPV). Additionally, a calibration plot with 5 risk groups was constructed to assess the agreement between the predicted probabilities and the actual observed outcomes. For multivariate analysis, variables with *p* < 0.1 in univariate analyses were entered into a binary logistic regression model using the forward likelihood ratio method, and odds ratios (ORs) with 95% confidence intervals (CIs) were calculated. Multicollinearity was assessed using the variance inflation factor (VIF), with values < 5 indicating the absence of significant multicollinearity. For multiple comparisons, *p* values were adjusted using the Bonferroni correction (e.g., pairwise comparison significance level α = 0.05 divided by the number of comparisons). A two-sided *p* < 0.05 was considered statistically significant.

### 2.4 Development of the combined predictive model

To integrate the complementary strengths of CTPA imaging parameters and serological markers, and to construct a comprehensive model for predicting high-risk APE, multivariable binary logistic regression analysis was conducted. Initially, variables with *p* values meeting the predefined threshold in the univariate analysis described in section 3.6.1 were included as candidate predictors. Variable selection was subsequently performed using the forward likelihood ratio method to identify independent predictors and establish the optimal model. Based on the finalized logistic regression equation, an individual predicted probability was calculated for each patient. This probability was defined as the composite score of the combined model, representing the overall likelihood of being classified as high-risk APE. The calculated probabilities were then used as a novel test variable in ROC curve analysis to assess the discriminative performance of the combined model for high-risk APE.

## 3 Results

### 3.1 Comparison of clinical characteristics between the control group and patients with APE stratified by risk

No statistically significant differences were identified among the five groups with respect to age, sex, smoking history, hypertension, prior stroke, diabetes, chest pain, or hemoptysis (all *p* > 0.05). However, the incidence of dyspnea was significantly higher in the intermediate-low-risk (94.1%) and intermediate-high-risk (92.9%) groups compared with the low-risk group (44.7%) and the control group (61.8%). Histories of alcohol consumption (case groups: 4.1%–21.4% vs. control group: 0%) and lower-extremity DVT (case groups: 57.1%–73.7% vs. control group: 9.0%) were significantly more frequent in the APE groups than in the control group (all *p* < 0.001). Detailed results are provided in **Table 1**.

**Table 1:** Comparison of clinical characteristics between the control group and patients with APE stratified by risk [n (%)/M (Q1, Q3)].

Variable	Control group (n = 89)	Low-risk group (n = 38)	Intermediate-low-risk group (n = 17)	Intermediate-high-risk group (n = 14)	High-risk group (n = 8)	Statistic	*p* value
Age (years)	63 (50.50, 72.00)	64.5 (53.00, 72.00)	68.0 (61.50, 73.50)	66.5 (53.00, 70.25)	63.0 (54.25, 70.25)	H=2.875	0.579
Male sex	48(53.9%)	22 (57.9%)	7 (41.2%)	6(42.9%)	3(37.5%)	–	0.637
Clinical symptoms							
Dyspnea	55(61.8%)	17(44.7%)	16(94.1%)	13(92.9%)	6(75.0%)	–	<0.001
Chest pain	23(25.8%)	16(42.1%)	9(52.9%)	4(28.6%)	4(50.0%)	–	0.098
Hemoptysis	18(20.2%)	8(21.1%)	5(29.4%)	2(14.3%)	1(12.5%)	–	0.866
Personal history							
Smoking history	33(37.1%)	10(26.3%)	4(23.5%)	4(28.6%)	3(37.5%)	–	0.703
Alcohol consumption	0(0.0%)	4(10.5%)	1(5.9%)	3(21.4%)	1(12.5%)	–	0.001
Past medical history							
Hypertension	28(31.5%)	14(36.8%)	8(47.1%)	5(35.7%)	0(0.0%)	–	0.186
Stroke	4(4.5%)	3 (7.9%)	2(11.8%)	2(14.3%)	0(0.0%)	–	0.343
Diabetes mellitus	0(0.0%)	6(15.8%)	3(17.6%)	0(0.0%)	0(0.0%)	–	0.435
Lower-extremity DVT	8(9.0%)	28(73.7%)	12(70.6%)	8(57.1%)	5(62.5%)	χ²=64.973	<0.001

### 3.2 Comparison of 256-detector-row CTPA findings among APE risk groups

#### 3.2.1 Comparison of thrombus distribution characteristics by risk stratification

Highly significant differences in thrombus distribution across pulmonary lobes and vascular levels were observed among the different risk stratification groups (all *p* < 0.001). As risk level increased, the proportion of bilateral pulmonary embolism rose from 44.7% in the low-risk group to 87.5% in the high-risk group. Similarly, the proportion of combined thrombosis involving both the main PA and its branches increased from 28.9% to 100%. Isolated branch thrombosis was predominant in the low-risk group (68.4%), whereas all patients in the high-risk group showed concomitant involvement of both the main PA and the branch vessels. Detailed findings are presented in **Table 2**.

**Table 2:** Comparison of thrombus distribution characteristics across the APE risk stratification groups [n (%)].

Thrombus distribution characteristics	Low-risk group (n = 38)	Intermediate-low-risk group (n = 17)	Intermediate-high-risk group (n = 14)	High-risk group (n = 8)	Statistic	*p* value
Pulmonary lobe					Fisher’s exact test	<0.001
Left lung only	4 (10.5)	2 (11.8)	1 (7.1)	0 (0.0)		
Right lung only	17 (44.7)	3 (17.6)	3 (21.4)	1 (12.5)		
Bilateral lungs	17 (44.7)	12 (70.6)	10 (71.4)	7 (87.5)		
Vessel level					Fisher’s exact test	<0.001
Branches only	26 (68.4)	9 (52.9)	4 (28.6)	0 (0.0)		
Main pulmonary artery only	1 (2.6)	0 (0.0)	3 (21.4)	0 (0.0)		
Main pulmonary artery + branches	11 (28.9)	8 (47.1)	7 (50.0)	8(100.0)		

#### 3.2.2 Pairwise comparisons of vascular involvement across risk stratification groups

Pairwise comparisons were conducted using Fisher’s exact test with Bonferroni correction (α = 0.0083). A statistically significant difference in the distribution of vascular involvement levels was observed only between the low-risk and high-risk groups (adjusted *p* < 0.001), while no significant differences were found among the remaining group comparisons (adjusted *p* > 0.0083). Detailed results are presented in **Supplementary Table 1**.

#### 3.2.3 Comparison of CTPA-derived imaging parameters across risk stratification groups

Significant differences in PA, PA/AO, RVD, LVD, and RVD/LVD were identified across the different risk stratification groups using the Kruskal–Wallis *H* test (all *p* < 0.05), whereas no significant differences were observed for AO and SVC measurements (all *p* > 0.05). With increasing risk stratification, the RVD/LVD ratio increased from 0.99 in the low-risk group to 1.55 in the intermediate-high-risk group and to 1.52 in the high-risk group. In contrast, LVD decreased from 39.72 mm in the low-risk group to 32.51 mm in the intermediate-high-risk group and to 34.41 mm in the high-risk group. Detailed results are presented in **Supplementary Table 2**.

#### 3.2.4 Pairwise comparisons of CTPA parameters across risk stratification groups

Pairwise comparisons were conducted using the Mann–Whitney U test with Bonferroni correction. Statistically significant differences in the RVD/LVD ratio were identified between the low-risk and intermediate-high-risk groups (adjusted *p* < 0.001), the low-risk and high-risk groups (adjusted *p* = 0.006), and the intermediate-low-risk and intermediate-high-risk groups (adjusted *p* < 0.001). A significant difference in RVD was observed only between the low-risk and intermediate-high-risk groups (adjusted *p* < 0.001). No statistically significant differences were found in PA or PA/AO measurements among the groups (adjusted *p* > 0.05). Detailed results are presented in **Supplementary Table 3**.

### 3.3 Comparison of D-dimer levels between the control group and patients with APE stratified by risk

The Kruskal–Wallis H test indicated statistically significant differences in D-dimer levels across the five groups (H = 45.593, *p* < 0.001). A progressive increase in D-dimer levels was observed with advancing risk stratification: the control group, 0.84 (0.51, 1.54) μg/mL; low-risk group, 1.99 (0.89, 2.78) μg/mL; intermediate-low-risk group, 2.00 (1.35, 4.16) μg/mL; intermediate-high-risk group, 3.05 (1.82, 4.05) μg/mL; and high-risk group, 4.80 (2.66, 7.61) μg/mL (**Table 3**).

**Table 3:** Comparison of D-dimer levels among groups [M (Q1, Q3), μg/mL].

Group	n	D-dimer	Statistic (H)	*p* value
Control group	89	0.84 (0.51, 1.54)	**H = 45.593**	**< 0.001**
Low-risk group	38	1.99 (0.89, 2.78)		
Intermediate-low-risk group	17	2.00 (1.35, 4.16)		
Intermediate-high-risk group	14	3.05 (1.82, 4.05)		
High-risk group	8	4.80 (2.66, 7.61)		

Data are expressed as median (lower quartile, upper quartile) [M (Q1, Q3)]. The H-value is the Kruskal-Wallis test statistic. P-values are used to assess the statistical significance of overall differences in D-dimer levels between groups.

Pairwise comparisons were conducted using the Mann–Whitney U test with Bonferroni correction (α = 0.005), revealing significantly higher D-dimer levels in all APE subgroups compared with the control group (adjusted *p* < 0.001). However, no statistically significant differences in D-dimer levels were found among the APE subgroups (adjusted *p* > 0.005) (**Supplementary Table 4**).

### 3.4 Correlation between D-dimer levels, CTPA parameters, and risk stratification

Spearman’s rank correlation analysis showed that the RVD/LVD ratio exhibited the strongest positive association with risk stratification (ρ=0.492; 95% CI: 0.30–0.64; p < 0.001), followed by RVD (ρ = 0.495; 95% CI: 0.31–0.65; *p* < 0.001), PA/AO (ρ = 0.389; 95% CI: 0.18–0.56; *p* < 0.001), PA (ρ = 0.381; 95% CI: 0.17–0.55; *p* < 0.001), and D-dimer levels (ρ = 0.351; 95% CI: 0.14–0.53; *p* = 0.002). In contrast, LVD was negatively correlated with risk stratification (ρ = −0.268; 95% CI: -0.46 to -0.05; p=0.019), while no significant correlations were observed for AO and SVC (both *p* > 0.05). Additionally, the RVD/LVD ratio showed a strong positive correlation with RVD (ρ = 0.606; 95% CI: 0.44–0.73; *p* < 0.001) and a strong negative correlation with LVD (ρ = −0.746; 95% CI: -0.83 to -0.63; *p* < 0.001), Other notable associations included a strong positive correlation between PA/AO and PA (ρ=0.752; 95% CI: 0.63–0.84; p<0.001) and a negative correlation between D-dimer and LVD (ρ=−0.421; 95% CI: -0.59 to -0.21; p<0.001). As presented in **Supplementary Table 5**.

### 3.5 Comparison of the predictive performance of individual indicators and combined models for high-risk APE

**Figure 2:**
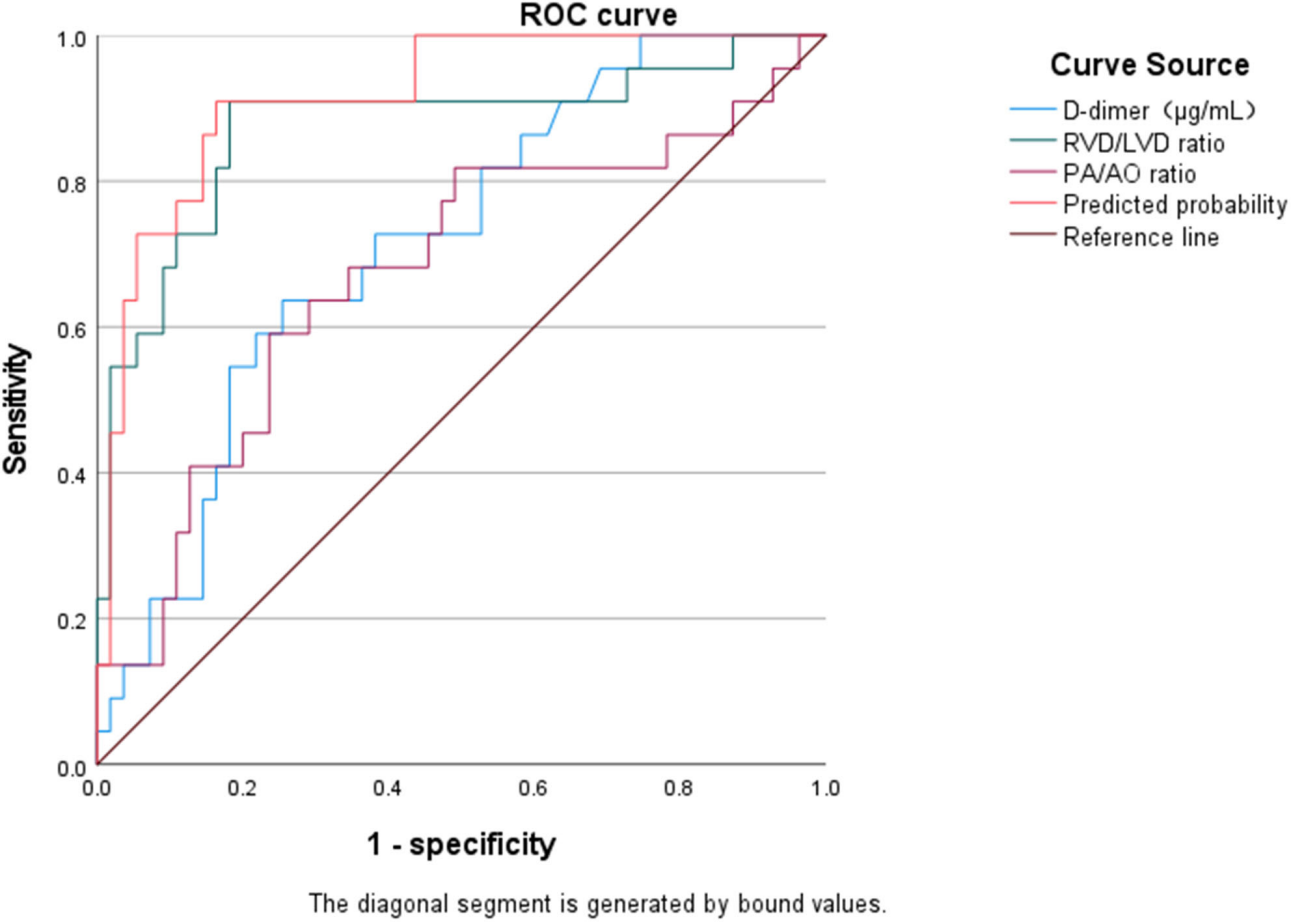
ROC curves of individual indicators and the combined model for predicting high-risk APE.

**Figure 3:**
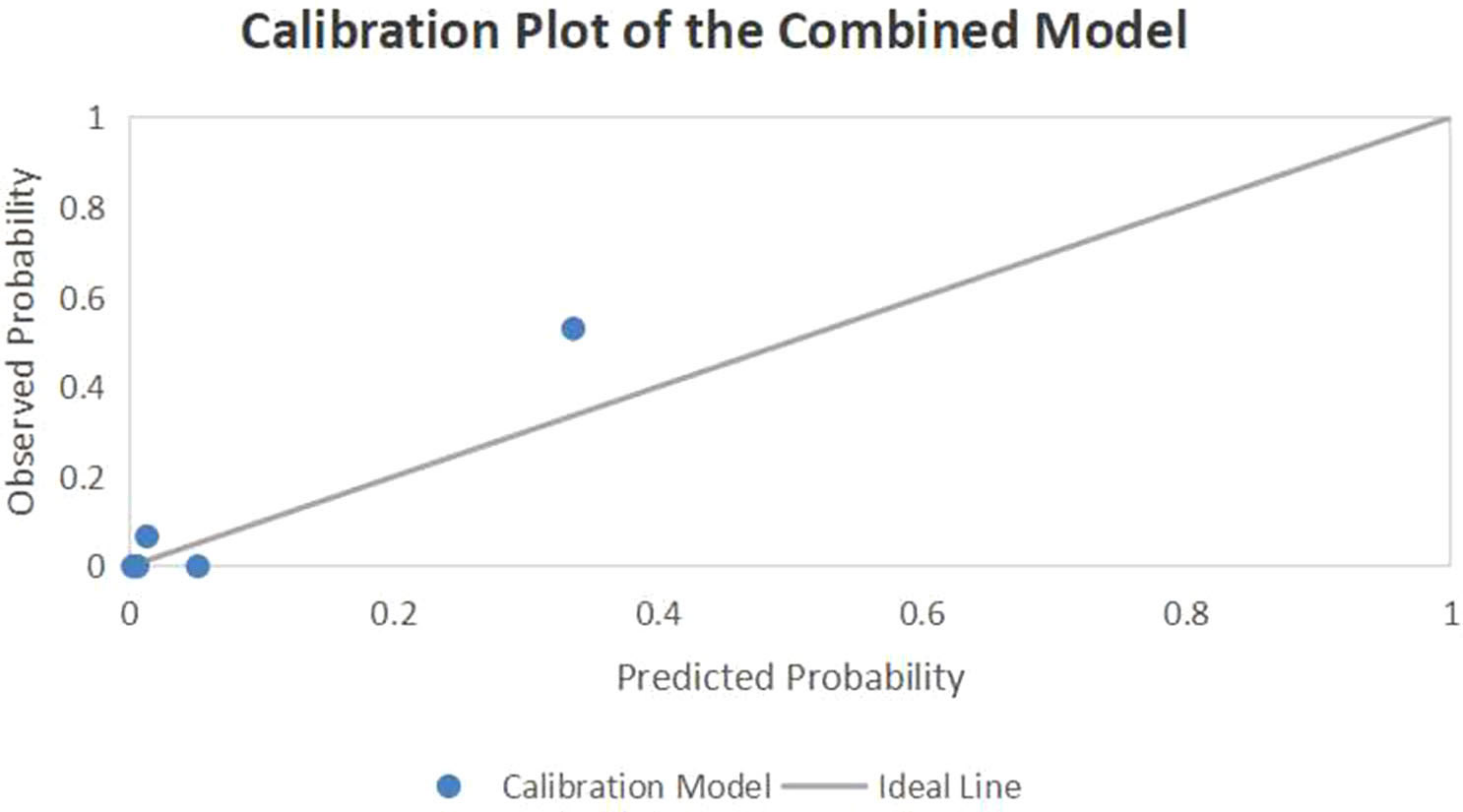
The calibration plot of the combined predictive model for high-risk APE.

ROC curve analysis showed that all individual indicators and combined models assessed yielded AUC values greater than 0.5, all of which were statistically significant (all *p* < 0.05). Among the individual indicators, the RVD/LVD ratio exhibited the highest predictive performance (AUC = 0.874, 95% CI: 0.773–0.976, *p* < 0.001), with an optimal cutoff value of 1.05, sensitivity of 81.8%, and specificity of 84.5%. The combined model incorporating RVD/LVD, PA/AO, and D-dimer demonstrated the highest discriminative capacity (AUC = 0.917, 95% CI: 0.852–0.983, *p* < 0.001), with an optimal cutoff value of 0.37, sensitivity of 86.4%, and specificity of 84.5% (**Figure 2** and **Table 4**). Furthermore, the calibration plot (**Figure 3**) demonstrated good agreement between the predicted and actual probabilities, indicating that the combined model has excellent calibration.

**Table 4:** Comparison of the predictive performance of individual indicators and the combined model for identifying high-risk APE.

Model/Indicator	AUC	Standard error	*p* value^a^	95% CI	Optimal cutoff	Sensitivity (%)	Specificity (%)	PPV (%)	NPV (%)
D-dimer	0.716	0.062	0.003	0.595–0.837	2.15μg/mL	81.8	58.6	40.9	90.1
RVD/LVD	0.874	0.052	<0.001	0.773–0.976	1.05	81.8	84.5	61.4	93.9
PA/AO	0.674	0.072	0.017	0.533–0.816	0.99	72.7	60.3	37.1	87.5
Combined model	0.917	0.034	<0.001	0.852–0.983	0.37	86.4	84.5	64.3	95.1

1. Definition of high-risk group: Patients classified as intermediate-high-risk (n = 14) and high-risk (n = 8) were grouped as the high-risk category (n = 22), while those classified as low-risk (n = 38) and intermediate-low-risk (n = 17) were grouped as the non-high-risk category (n = 55). ^a^*P* value tests the null hypothesis that the true AUC is equal to 0.5. Optimal cutoff value, sensitivity, and specificity were determined based on the maximum Youden index. PPV and NPV were calculated using the prevalence of high-risk APE in this cohort (28.57%).

### 3.6 Analysis of independent risk factors for poor prognosis

#### 3.6.1 Univariate analysis of baseline characteristics between the favorable and poor prognosis groups

Among the 77 patients with APE, 10 (12.99%) were classified into the poor-prognosis group and 67 (87.01%) into the favorable-prognosis group. Univariate analysis indicated that PA (32.35 vs. 28.84 mm), RVD (47.23 vs. 40.01 mm), RVD/LVD (1.62 vs. 1.03), and PA/AO (0.98 vs. 0.83) were significantly higher in the poor-prognosis group, while LVD was significantly lower (29.72 vs. 39.14 mm) (all *p* < 0.1). Additionally, the proportion of thrombus involving the main PA or both the main and branch pulmonary arteries was markedly greater in the poor-prognosis group compared with the favorable-prognosis group (80.0% vs. 44.8%, *p* < 0.1). No statistically significant differences were observed between the two groups in age, sex, clinical manifestations, personal history, past medical history, or D-dimer levels (all *p* > 0.1), as summarized in **Table 5**.

**Table 5:** Univariate analysis of baseline characteristics between the favorable and poor prognosis groups.

Variable	Overall (n = 77)	Favorable prognosis (n = 67)	Poor prognosis (n = 10)	Statistic	*p* value
Age [years, M (Q1, Q3)]	66.0 (54.50, 71.50)	66.0 (54.0, 72.0)	62.00(54, 68.75)	U=293	0.524
Male sex [n (%)]	38(49.4)	34(50.7)	4(40.0)	–	0.737
Clinical symptoms [n (%)]					
Dyspnea	52 (67.5)	44(65.7)	8 (80.0)	–	0.485
Chest pain	33 (42.9)	30 (44.8)	3 (30)	–	0.502
Hemoptysis	16(20.8)	15(22.4)	1 (10.0)	–	0.678
Personal history [n (%)]					
Smoking history	21 (27.3)	18(34.8)	3(30.0)	–	1.000
Alcohol consumption	9 (11.7)	6 (9.0)	3 (30.0)	–	0.088
Past medical history [n (%)]					
Hypertension	27 (35.1)	26 (38.8)	1 (10.0)	–	0.090
Stroke	7 (9.1)	7 (10.4)	0(0)	–	0.585
Diabetes mellitus	9(11.7)	9 (13.4)	0(0)	–	0.596
Lower-extremity DVT	53 (68.8)	49(73.1)	4(40.0)	–	0.062
D-dimer (μg/mL), M (Q1, Q3)	1.50 (0.80, 3.07)	2.01 (1.33, 3.43)	3.07(1.96, 5.55)	U=457	0.064
Imaging characteristics, n (%)					
Thrombus location					
Bilateral lungs	46 (59.7)	39 (58.2)	7 (70)		0.78
Right lung	24 (31.2)	22 (32.8)	2 (20)		
Left lung	7 (9.1)	6 (9.0)	1 (10)		
Vessel level [n (%)]					
Main pulmonary artery	4 (5.2)	2 (3.0)	2 (20)	–	0.022
Branches	39 (50.6)	37 (55.2)	2 (20)		
Main pulmonary artery + branches	34 (44.2)	28(41.8)	6(60)		
CTPA parameters (mm), M (Q1, Q3)					
PA (mm)	29.22 (26.42, 32.41)	28.84 (26.09, 31.62)	32.35 29.30, 40.53)	U=495.5	0.015 ​
AO (mm)	35.57 (32.31, 37.5)	35.67 (32.22, 37.60)	35.07 (32.51, 37.12)	U=312.5	0.733
PA/AO​	0.83 (0.74, 0.97)	0.83 (0.73, 0.97)	0.98 (0.82, 1.17)	U=478.0	0.030
RVD (mm)	42.08 (36.43, 48.98)	40.01 (35.05, 47.88)	47.23 (43.54, 54.83)	U=506	0.010​
LVD (mm)	37.98(31.8, 43.87)	39.14 (33.19, 44.10)	29.72 (23.16, 38.94)	U=177	0.017
RVD/LVD	1.05(0.91, 1.43)	1.03 (0.91, 1.28)	1.62 (1.40, 1.96)	U=575	<0.001​
SVC (mm)​	22.97 (20.96, 25.80)	22.92 (20.93, 25.39)	24.04 (22.71, 27.36)	U=427	0.163

#### 3.6.2 Multivariate logistic regression analysis of factors associated with poor prognosis in patients with APE

Variables with *p* < 0.1 in the univariate analysis, including history of alcohol consumption, hypertension, history of lower-extremity DVT, D-dimer, vessel level, PA, PA/AO, RVD, LVD, and RVD/LVD, were entered into the multivariate logistic regression model. No evidence of significant multicollinearity was identified, as all VIF values were < 5. The analysis identified RVD/LVD (odds ratio [OR] = 124.605, 95% confidence interval [CI]: 6.960–2230.719, *p* = 0.001) and D-dimer (OR = 1.313, 95% CI: 1.024–1.685, *p* = 0.032) as independent risk factors for poor prognosis. Although a history of lower-extremity DVT showed a trend toward significance, it did not reach the threshold for statistical significance (OR = 5.908, 95% CI: 0.920–37.940, *p* = 0.061), as presented in **Table 6**.

**Table 6:** Multivariate logistic regression analysis of factors associated with poor prognosis in patients with APE.

Variable	β value	Standard error	Wald χ² value	*p* value	OR value	95% CI	VIF
RVD/LVD ratio	4.825	1.472	10.746	0.001	124.605	6.960-2230.719	1.85
D-dimer (μg/mL)	0.273	0.127	4.602	0.032	1.313	1.024-1.685	1.23
History of lower-extremity DVT (yes)	1.776	0.949	3.504	0.061	5.908	0.920-37.94	1.18
Constant	-10.288	2.650	15.074	<0.001	–	–	–

Variable selection was performed using the forward likelihood ratio method.

## 4 Discussion

### 4.1 Relationship between clinical characteristics, risk stratification, and prognosis in APE

Exertional dyspnea and shortness of breath are the most common clinical manifestations of APE, whereas the classic triad of pulmonary infarction chest pain, hemoptysis, and dyspnea occurs less frequently (Kaptein et al., 2021). In this study, the incidence of dyspnea in the high-risk group (75.0%) was unexpectedly lower than that observed in the intermediate-high-risk (92.9%) and intermediate-low-risk (94.0%) groups. This finding may be attributable to the limited sample size; therefore further investigation in larger cohorts is warranted. The comparatively lower incidence of dyspnea in the low-risk group (44.7%) and the control group (61.8%) indicates that, although dyspnea is an important clinical indicator of APE, accurate risk stratification necessitates a comprehensive assessment incorporating both imaging findings and biomarker profiles.

Lower-extremity DVT is recognized as the primary source of emboli in APE (Pulmonary Embolism & Pulmonary Vascular Diseases Group of the Chinese Thoracic Society et al., 2025). The prevalence of a prior history of lower-extremity DVT among patients with APE (57.1%–73.7%) was significantly higher than that observed in the control group (9.0%), supporting the use of lower-extremity vascular ultrasound as a routine screening modality in populations at elevated risk for APE. This finding was consistent with those reported by Giordano et al. and Meignan et al (Meignan et al., 2000; Giordano et al., 2017). A history of alcohol consumption varied significantly among patients with APE (*p* = 0.001), which may be related to alcohol-induced coagulation abnormalities and endothelial dysfunction. Prospective studies are needed to further clarify the dose–response relationship between alcohol intake and APE risk.

### 4.2 Relationship between 256-detector-row CTPA imaging features and risk stratification in APE

The high spatial and temporal resolution of 256-detector-row helical CT allows for accurate delineation of thrombus location, evaluation of PA dilation, and assessment of right ventricular function (Zhao et al., 2023). Direct imaging signs include intraluminal contrast-filling defects within the pulmonary arteries, while indirect signs consist of pleural-based wedge-shaped opacities, mosaic perfusion patterns, discoid atelectasis, central PA dilation, and reduced attenuation of distal vascular branches. Incidental findings, potential diagnostic pitfalls, and modality-specific imaging artifacts should be carefully considered during the interpretation of CTPA studies (Bukhari et al., 2025). In this study, higher risk stratification was associated with a significant rise in the frequency of bilateral pulmonary embolism and combined involvement of the main PA and branch vessels. Notably, combined main PA and branch thrombosis was observed in 100% of patients classified as high risk, indicating that this imaging feature may represent a potential marker of high-risk APE. However, due to the limited number of high-risk cases (n = 8), confirmation in larger cohorts is necessary.

Among the CTPA-derived parameters, the RVD/LVD ratio is a key metric for risk stratification in APE, indicating a strong positive correlation with risk stratification (ρ = 0.492, *p* < 0.01) and effectively distinguishing between intermediate-low-risk and intermediate-high-risk patients (adjusted *p* < 0.001), consistent with the findings reported by Zhu et al (Zhu et al., 2022). From a pathophysiological standpoint, mechanical obstruction involving approximately 30%–50% of the pulmonary vascular bed by thrombi leads to elevated pulmonary vascular resistance, resulting in an acute increase in right ventricular afterload (Lyhne et al., 2021). This hemodynamic burden alters right ventricular myocardial contractility via the Frank–Starling mechanism and induces compensatory right ventricular dilation (elevated RVD). The resulting increase in right ventricular wall tension and myocyte stretch prolongs right ventricular contraction. Concurrent right ventricular dilation displaces the interventricular septum toward the left (septal shift), impairs left ventricular filling (reduced LVD), and ultimately increases the RVD/LVD ratio, which directly reflects the severity of right ventricular dysfunction (Watts et al., 2010; Alerhand and Adrian, 2023). Becattini et al. identified right ventricular dysfunction as a major contributor to increased short-term mortality in APE, and quantification using the RVD/LVD ratio was shown to provide superior prognostic value (Becattini et al., 2021). Khalid M et al. proposed that the PA/AO ratio may serve as an indicator of pulmonary hypertension severity, based on the association between elevated PA pressure and increased PA diameter (Khalid et al., 2019).

A Reduction in aortic diameter may further elevate the PA/AO ratio, thereby reflecting disease progression. In this study, although both PA and PA/AO demonstrated positive correlations with risk stratification, pairwise comparisons did not yield statistically significant differences between groups. This finding may be explained by considerable interindividual anatomical variability affecting PA measurements and the susceptibility of the PA/AO ratio to confounding factors such as age and blood pressure. No significant differences in SVC measurements were observed among risk groups, indicating limited use for APE risk stratification, potentially due to the influence of intrathoracic pressure, circulating blood volume, and other hemodynamic variables.

### 4.3 The value of D-dimer in the assessment of APE

D-dimer is a sensitive biomarker of systemic fibrinolytic activity. In this study, D-dimer concentrations were significantly higher in all APE patients compared with controls, supporting its diagnostic use in excluding APE. This observation is consistent with findings reported by Feng et al., who noted a diagnostic sensitivity exceeding 90% (Feng and Wu, 2021). However, consistent with the notion that D-dimer reflects thrombotic burden rather than hemodynamic compromise, our results showed no statistically significant difference in D-dimer levels among the APE risk subgroups. D-dimer alone cannot effectively differentiate among APE risk categories, as it primarily reflects activation of fibrinolysis and clot load rather than the direct determinants of hemodynamic severity. Risk stratification in APE is influenced by multiple parameters, including thrombus burden, right ventricular function, and evidence of myocardial injury. Therefore, reliance on a single serological marker is insufficient to capture the multidimensional nature of APE severity (Konstantinides et al., 2020). While D-dimer serves as a valuable tool for excluding APE in low-risk individuals (e.g., levels <0.5 μg/mL), its utility in predicting prognosis or grading clinical severity is limited when used in isolation. Instead, it should be interpreted as a complementary marker that adds information on thrombotic burden to the assessment of hemodynamic function. In clinical settings, APE may be reasonably excluded in individuals with suspected disease and D-dimer levels below 0.5 μg/mL, thereby reducing the need for unnecessary CTPA examinations, minimizing radiation exposure, and lowering overall healthcare costs (Feng and Wu, 2021).

### 4.4 Clinical significance and potential applications of the combined predictive model

In this study, a combined predictive model incorporating CTPA-derived imaging parameters and serological markers was developed and validated. The primary advantage of this model lies in its capacity to assess APE from structural, functional, and molecular perspectives. Specifically, the RVD/LVD ratio serves as a quantitative indicator of right ventricular dilation and dysfunction resulting from acute pulmonary hypertension, providing a direct morphological assessment of hemodynamic impairment. D-dimer levels function as a sensitive marker of thrombus burden and the associated hyperfibrinolytic state. The complementary integration of these parameters allows the model to reflect two principal pathophysiological processes contributing to adverse outcomes in APE: mechanical vascular obstruction and neurohumoral activation. This multidimensional approach offers a more comprehensive and precise method for risk assessment compared with models relying on a single parameter.

The results of the multivariate logistic regression analysis further support the biological plausibility of the proposed predictive model. Both the RVD/LVD ratio (odds ratio [OR] = 124.605) and D-dimer levels (OR = 1.313) were identified as independent risk factors for high-risk classification among patients with APE. These findings have direct clinical relevance. The RVD/LVD ratio emerged as the strongest predictor of high-risk status, underscoring the central role of right ventricular dysfunction in risk stratification. Additionally, the independent prognostic contribution of D-dimer highlights the importance of incorporating thrombotic burden alongside hemodynamic impairment in comprehensive risk assessment.

The association between elevated D-dimer levels and thrombus burden is consistent with previous reports, such as the study by Keller et al (Keller et al., 2018), which demonstrated a significant correlation between D-dimer concentration and thrombus burden categories. Notably, Keller et al. (2018) further proposed that D-dimer >1.18 mg/L could serve as an independent predictor for RVD in normotensive patients. In contrast, our study found that while D-dimer remained an independent predictor, the RVD/LVD ratio was a substantially stronger determinant of high-risk status (OR = 124.605 vs. OR = 1.313). This discrepancy may be attributed to the direct anatomical and functional correlation of the RVD/LVD ratio with hemodynamic compromise, whereas D-dimer reflects the global thrombotic burden without specific localization.

From the optimal cutoff values identified in this study (RVD/LVD ≥ 1.05 and D-dimer ≥ 2.15 μg/mL), along with the high NPV of the combined model (95.1%), several potential applications may be hypothesized. Furthermore, the calibration plot demonstrated good agreement between the predicted and observed probabilities of high-risk APE, with data points closely following the ideal line, suggesting that the model possesses not only strong discriminative ability (AUC = 0.917) but also excellent calibration in our internal validation cohort. However, it is crucial to note that the strong discriminative performance observed (AUC = 0.917) may be optimistic due to the single-center, retrospective design and the lack of external validation. Therefore, the current findings should be regarded as exploratory rather than definitive. While the model shows promise as an adjunctive tool to support clinicians in identifying patients at high risk in future validations, its routine clinical implementation is premature. Importantly, if validated in broader cohorts, the high NPV could theoretically support strategies for identifying patients at very low risk of short-term mortality. Further multicenter prospective studies are warranted to confirm whether such applications can truly facilitate efficient resource allocation and reduce healthcare burden without compromising patient safety.

### 4.5 Study limitations and future directions

Several limitations should be acknowledged. First, the retrospective study design may have introduced selection bias, and some confounding variables could not be fully controlled. Second, the overall sample size was limited, particularly within the high-risk group (n = 8) and the poor-prognosis group (n = 10), which may have contributed to an overestimation of ORs in the multivariate analysis and restricted the generalizability of the proposed model. Third, only D-dimer and CTPA-derived parameters were included in the predictive model; established biomarkers with recognized value in APE risk stratification, such as troponin and B-type natriuretic peptide (BNP), were not incorporated, thereby limiting the comprehensiveness of the assessment. The short-term follow-up period of 30 days did not permit assessment of long-term prognostic outcomes, such as 1-year survival or recurrent embolic events. Finally, as the data were obtained from a single center and no external validation cohort was included, the reported AUC of 0.917 may be inflated due to overfitting and selection bias specific to our institution. The model’s generalizability to other populations with different demographic or clinical characteristics remains uncertain. Consequently, further studies in larger, multicenter populations with external validation are needed to confirm the true predictive performance and robustness of the model before any clinical recommendations can be made.

Future investigations should aim to address these limitations through several key approaches. First, prospective, multicenter studies with larger cohorts are needed to validate the robustness and generalizability of the combined predictive model. Second, systematic incorporation of additional biomarkers, such as troponin, BNP, and microRNAs may support the development of a multidimensional predictive framework encompassing imaging, metabolic, and functional domains, thereby improving the precision of risk assessment. Third, the high-resolution capabilities of 256-detector-row helical CTPA merit further investigation, particularly with respect to emerging imaging indicators such as perfusion metrics and quantitative assessment of thrombus burden. Finally, extension of the follow-up period is warranted to assess the predictive value of the model for intermediate- and long-term outcomes, including quality of life and hospital readmission rates. Such studies should incorporate health economic evaluations to determine the clinical applicability and cost-effectiveness of the model.

## 5 Conclusion

This study systematically assessed 256-detector-row helical CTPA parameters in combination with D-dimer to construct a predictive model for risk stratification and prognostic assessment in APE. The results demonstrated that the RVD/LVD ratio functions as a key imaging marker for quantifying right ventricular dysfunction and identifying patients at high risk, while integration with D-dimer yielded excellent predictive performance (AUC = 0.917). The model facilitates a multimodal assessment within the structural–functional framework, providing an objective and quantitative basis for early risk stratification and informed therapeutic decision-making in patients with APE by incorporating both imaging and serological data. While the required parameters are accessible and reproducible, the model currently serves as a promising exploratory tool rather than a clinical standard. Given the limitations of our single-center retrospective design, broad clinical implementation across various levels of healthcare is not yet recommended. Future multicenter validation is essential to establish its robustness. Once validated, the model has the potential to contribute to more standardized and precise management of APE.

## Data Availability

The original contributions presented in the study are included in the article/**Supplementary Material**. Further inquiries can be directed to the corresponding author.
