# Supplementary material for: Prognostic utility of combined 256-detector-row computed tomographic pulmonary angiography and D-dimer in risk stratification of acute pulmonary embolism: a retrospective cohort analysis
Source: Front Physiol. 2026 Apr 10;17:1796105. doi: 10.3389/fphys.2026.1796105 (PMC13105458; doi:10.3389/fphys.2026.1796105)
Supplement: Supplementary file 1 [file Table1.docx]

**Supplementary Table 1: Pairwise** **comparisons** of **vascular involvement levels across APE risk stratification groups**

| **Comparison** | ***p* value (Fisher’s exact test)** | **Adjusted *p* value (Bonferroni)** | **Significance** |
| --- | --- | --- | --- |
| Low-risk vs. intermediate-low-risk | 0.473 | >1.000 | No |
| Low-risk vs. intermediate-high-risk | 0.011 | 0.066 | No |
| Low-risk vs. high-risk | <0.001 | <0.001 | Yes |
| intermediate-low-risk vs. intermediate-high-risk | 0.103 | 0.618 | No |
| intermediate-low-risk vs. high-risk | 0.022 | 0.132 | No |
| intermediate-high-risk vs. high-risk | 0.083 | 0.498 | No |

**Supplementary Table 2: Comparison of CTPA-derived imaging parameters across APE risk stratification groups [M (Q1, Q3), mm]**

| **Parameter** | **Low-risk group (n = 38)** | **Intermediate-low-risk group (n = 17)** | **Intermediate-high-risk group (n = 14)** | **High-risk group (n = 8)** | **H value** | ***p* value** |
| --- | --- | --- | --- | --- | --- | --- |
| PA | 27.48 (24.93, 30.15) | 31.62 (27.91, 35.96) | 31.25 (29.03, 35.83) | 29.78 (26.37, 35.13) | 14.918 | 0.002 |
| AO | 35.62 (32.80, 37.66) | 36.33 (31.31, 37.85) | 35.92 (32.96, 37.12) | 32.43 (28.18, 35.17) | 3.755 | 0.289 |
| PA/AO | 0.78 (0.70, 0.89) | 0.92 (0.77, 1.07) | 0.87 (0.82, 1.03) | 1.02 (0.78, 1.10) | 12.321 | 0.006 |
| RVD | 37.77 (33.34, 43.58) | 40.01 (36.73, 49.86) | 48.22 (45.59, 54.28) | 44.55 (43.33, 56.00) | 20.083 | <0.001 |
| LVD | 39.72 (33.68, 44.72) | 40.22 (34.49, 46.32) | 32.51 (28.24, 38.57) | 34.41 (23.48, 39.64) | 9.981 | 0.019 |
| RVD/LVD | 0.99 (0.88, 1.12) | 1.01 (0.87, 1.09) | 1.55 (1.30, 1.74) | 1.52 (1.27, 1.72) | 26.093 | <0.001 |
| SVC | 22.75 (20.85, 25.27) | 22.65 (21.09, 25.61) | 23.31 (21.63, 25.20) | 25.46 (21.23, 27.48) | 2.288 | 0.515 |

Note: PA, pulmonary artery diameter; AO, aortic diameter; RVD, right ventricular diameter; LVD, left ventricular diameter; SVC, superior vena cava diameter.

**Supplementary Table 3: Pairwise comparisons of CTPA-derived imaging parameters across APE risk stratification groups**

| **CTPA Parameter** | **Comparison** | **Mann–Whitney U** | ***p* value** | **Adjusted *p* value** | **Significance** |
| --- | --- | --- | --- | --- | --- |
| PA (mm) | Low-risk vs. intermediate-low-risk | 156.000 | 0.002 | 0.012 | No |
|  | Low-risk vs. intermediate-high-risk | 116.000 | 0.002 | 0.012 | No |
|  | Low-risk vs. high-risk | 100.000 | 0.138 | 0.828 | No |
|  | Intermediate-low-risk vs. intermediate-high-risk | 115.500 | 0.891 | 1.000 | No |
|  | Intermediate-low-risk vs. high-risk | 56.000 | 0.511 | 1.000 | No |
|  | Intermediate-high-risk vs. high-risk | 43.000 | 0.402 | 1.000 | No |
| PA/AO | Low-risk vs. intermediate-low-risk | 182.000 | 0.010 | 0.06 | No |
|  | Low-risk vs. intermediate-high-risk | 143.000 | 0.011 | 0.066 | No |
|  | Low-risk vs. high-risk | 75.000 | 0.025 | 0.15 | No |
|  | Intermediate-low-risk vs. intermediate-high-risk | 119.000 | 1.000 | 1.000 | No |
|  | Intermediate-low-risk vs. high-risk | 57.000 | 0.549 | 1.000 | No |
|  | Intermediate-high-risk vs. high-risk | 46.000 | 0.525 | 1.000 | No |
| RVD (mm) | Low-risk vs. intermediate-low-risk | 238.000 | 0.122 | 0.732 | No |
|  | Low-risk vs. intermediate-high-risk | 82.000 | <0.001 | <0.001 | Yes |
|  | Low-risk vs. high-risk | 48.000 | 0.002 | 0.012 | No |
|  | Intermediate-low-risk vs. intermediate-high-risk | 63.000 | 0.026 | 0.156 | No |
|  | Intermediate-low-risk vs. high-risk | 37.500 | 0.075 | 0.45 | No |
|  | Intermediate-high-risk vs. high-risk | 49.000 | 0.664 | 1.000 | No |
| RVD/LVD | Low-risk vs. intermediate-low-risk | 320.000 | 0.956 | 1.000 | No |
|  | Low-risk vs. intermediate-high-risk | 63.000 | <0.001 | <0.001 | Yes |
|  | Low-risk vs. high-risk | 41.000 | 0.001 | 0.006 | Yes |
|  | Intermediate-low-risk vs. intermediate-high-risk | 30.000 | <0.001 | <0.001 | Yes |
|  | Intermediate-low-risk vs. high-risk | 18.000 | 0.002 | 0.012 | No |
|  | Intermediate-high-risk vs. high-risk | 54.000 | 0.920 | 1.000 | No |

Note: Only pairwise comparisons of CTPA parameters depicting statistical significance (*p* < 0.05) in the Kruskal–Wallis test (Table 4) are presented.

**Supplementary Table 4: Pairwise comparisons of D-dimer levels following Bonferroni correction**

| **Comparison** | **Mann–Whitney U** | ***p* value** | **Adjusted *p* value** | **Significance** |
| --- | --- | --- | --- | --- |
| Control vs. low-risk | 974.000 | <0.001 | <0.001 | **Yes** |
| Control vs. Intermediate-low-risk | 324.000 | <0.001 | <0.001 | **Yes** |
| Control vs. Intermediate-high-risk | 164.500 | <0.001 | <0.001 | **Yes** |
| Control vs. high-risk | 57.500 | <0.001 | <0.001 | **Yes** |
| Low-risk vs. Intermediate-low-risk | 277.500 | 0.407 | >1.000 | **No** |
| Low-risk vs. Intermediate-high-risk | 165.5 | 0.038 | 0.380 | **No** |
| Low-risk vs. high-risk | 56.000 | 0.004 | 0.040 | **No** |
| Intermediate-low-risk vs. Intermediate-high-risk | 90.000 | 0.262 | >1.000 | **No** |
| Intermediate-low-risk vs. High-risk | 32.500 | 0.037 | 0.370 | **No** |
| Intermediate-high-risk vs. high-risk | 32.000 | 0.110 | >1.000 | **No** |

**Supplementary Table 5. Spearman’s Correlation Coefficients between D-dimer Levels, CTPA-Derived Parameters, and Risk Stratification in Acute Pulmonary Embolism(N=77)**

| **Variable** | **Spearman's ρ** | **95% Confidence Interval** | **p-value** |
| --- | --- | --- | --- |
| **Correlations with Risk Stratification** | |  |  |
| RVD/LVD Ratio | 0.492** | (0.30, 0.64) | < 0.001 |
| RVD​ | 0.495** | (0.31, 0.65) | < 0.001 |
| PA/AO​ | 0.389** | (0.18, 0.56) | < 0.001 |
| PA​ | 0.381** | (0.17, 0.55) | < 0.001 |
| D-dimer | 0.351* | (0.14, 0.53) | 0.002 |
| LVD | -0.268* | (-0.46, -0.05) | 0.019 |
| AO | -0.112 | (-0.33, 0.11) | 0.332 |
| SVC | -0.085 | (-0.30, 0.14) | 0.461 |
| **Inter-correlations Among Key Parameters** | |  |  |
| RVD/LVD vs. RVD | 0.606** | (0.44, 0.73) | < 0.001 |
| RVD/LVD vs. LVD | -0.746** | (-0.83, -0.63) | < 0.001 |
| PA vs. RVD | 0.441** | (0.24, 0.60) | <0.001 |
| PA/AO vs. PA | 0.752** | (0.63, 0.84) | <0.001 |
| PA/AO vs. AO | -0.543** | (-0.69, -0.35) | <0.001 |
| D-dimer vs. LVD | -0.421** | (-0.59, -0.21) | <0.001 |

Note: Data are presented as Spearman’s rank correlation coefficients (ρ). 95% confidence intervals (CI) were calculated using the Bootstrap method .with 1,000 resamples *p < 0.05, **p < 0.01 (two-tailed).
